# Supplementary material for: Predicting antimicrobial resistance in Pseudomonas aeruginosa with machine learning‐enabled molecular diagnostics
Source: EMBO Mol Med. 2020 Feb 12;12(3):e10264. doi: 10.15252/emmm.201910264 (PMC7059009; doi:10.15252/emmm.201910264)

Predicting antimicrobial resistance in *Pseudomonas aeruginosa* with machine learning-enabled molecular diagnostics

Ariane Khaledi, Aaron Weimann, Monika Schniederjans, Ehsaneddin Asgari, Tzu-Hao Kuo, Antonio Oliver, Gabriel Cabot, Axel Kola, Petra Gastmeier, Michael Hogardt, Daniel Jonas, Mohammad R.K. Mofrad, Andreas Bremges, Alice C. McHardy, Susanne Häussler

Appendix

Table of Contents

Appendix Figure S1
Appendix Figure S2
Appendix Figure S3

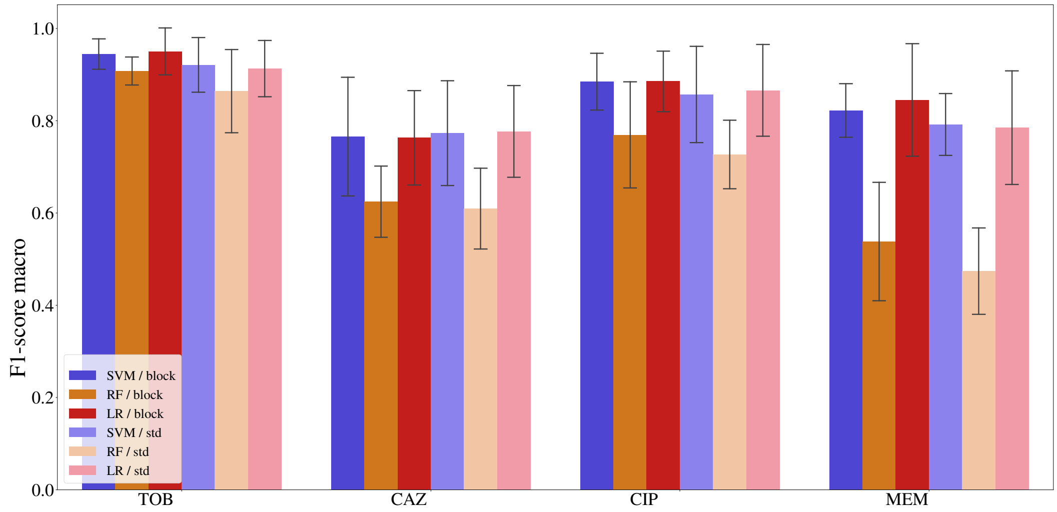

Appendix Figure S1: Comparison of support vector machine, random forest, and logistic regression classifiers in antimicrobial resistance prediction, and their generalization ability. Training and performance estimates was performed in ten-fold cross-validation, where isolates were split either randomly (standard: std) or using phylogenetically related blocks of isolates (block). The classification was performed for each antibiotic using the best performing feature combinations for the SVM. The error bar shows the variability (standard deviation) in 10 test folds.

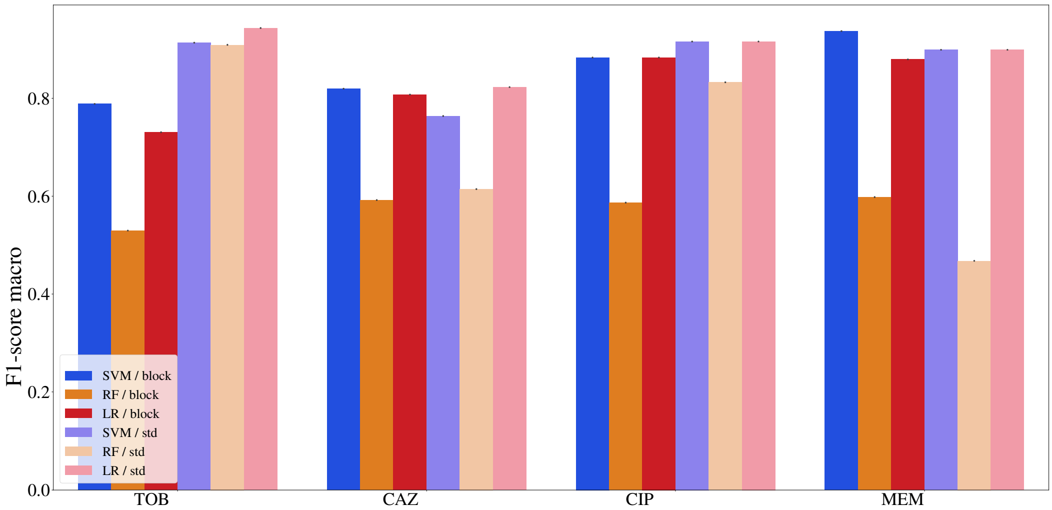

Appendix Figure S2: Comparison of support vector machine, random forest, and logistic regression classifiers in antimicrobial resistance prediction, and their generalization ability. The classifiers were tuned in a ten-fold cross-validation; subsequently their performances are reported over the held-out set, where isolates were split either randomly (standard: std) or using phylogenetically related blocks of isolates (block).

Appendix Figure S3: Pearson correlation coefficient of independently sequenced PA14-wt samples.

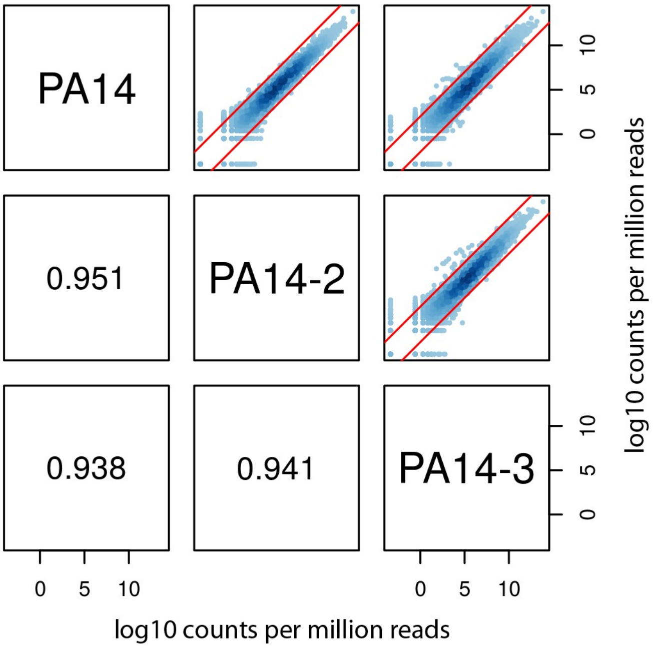

Supplement: Supplementary file 1 — Appendix [file EMMM-12-e10264-s001.pdf]
