# Supplementary material for: Predicting antimicrobial resistance in Pseudomonas aeruginosa with machine learning‐enabled molecular diagnostics
Source: EMBO Mol Med. 2020 Feb 12;12(3):e10264. doi: 10.15252/emmm.201910264 (PMC7059009; doi:10.15252/emmm.201910264)
Supplement: Supplementary file 2 — Expanded View Figures PDF [file EMMM-12-e10264-s002.pdf]

Expanded View Figures

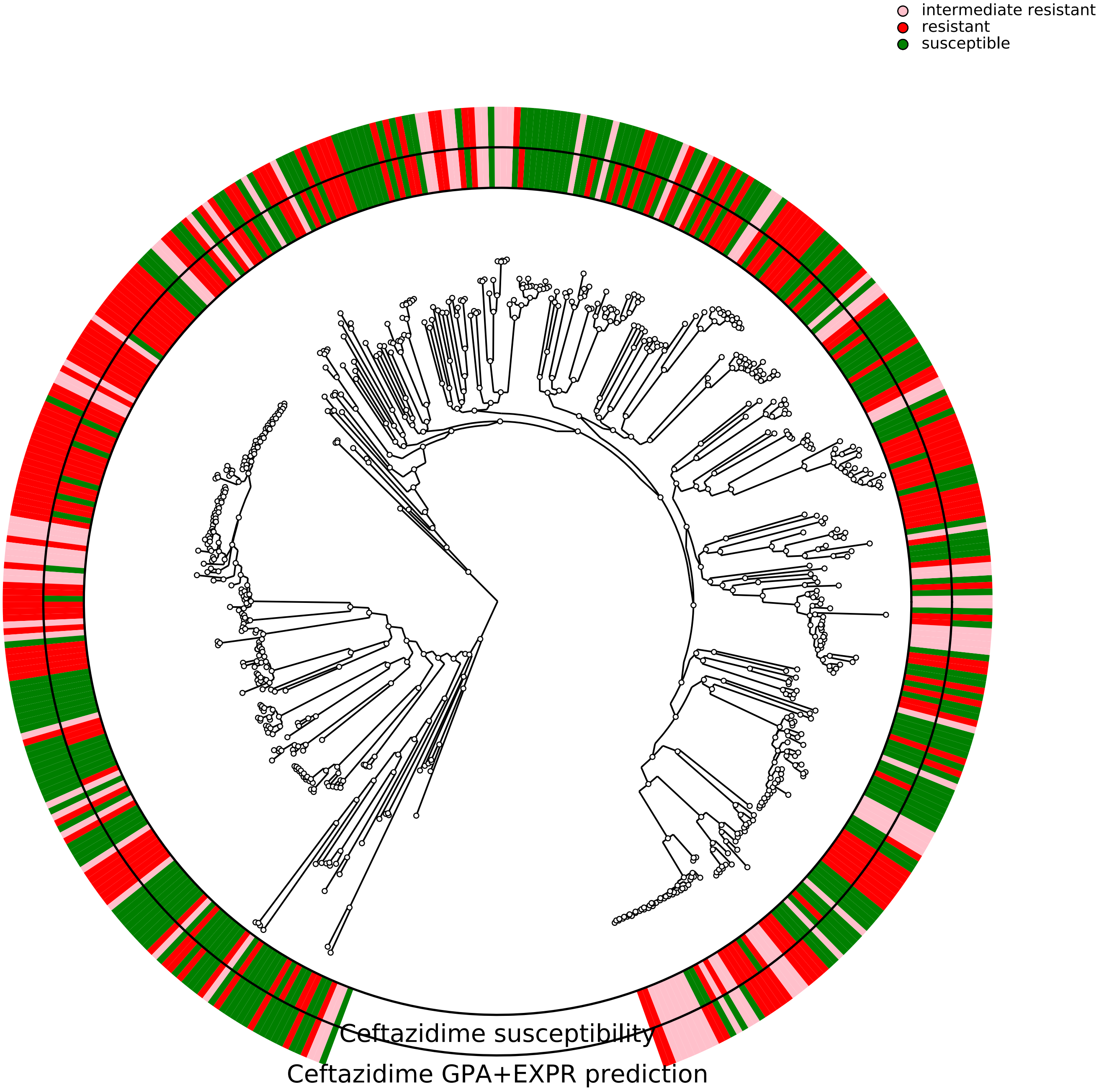

Figure EV1. Correlation of susceptibility profiling by MIC testing and resistance prediction using diagnostic classifiers for ciprofloxacin.

The phylogenetic tree is based on the 414 *Pseudomonas aeruginosa* isolates used in this study. The branches leading toward two deeply branching clades were collapsed (CH4433 and ESP077, and CH4684, CH5206, and CH5387). The inner ring depicts the susceptibility of each isolate to ciprofloxacin; green, susceptible; red, resistant; rose, intermediate resistant. The outer rings show the susceptibility as assigned by the diagnostic classifier (intermediate resistant samples were not assigned).

© 2020 The Authors

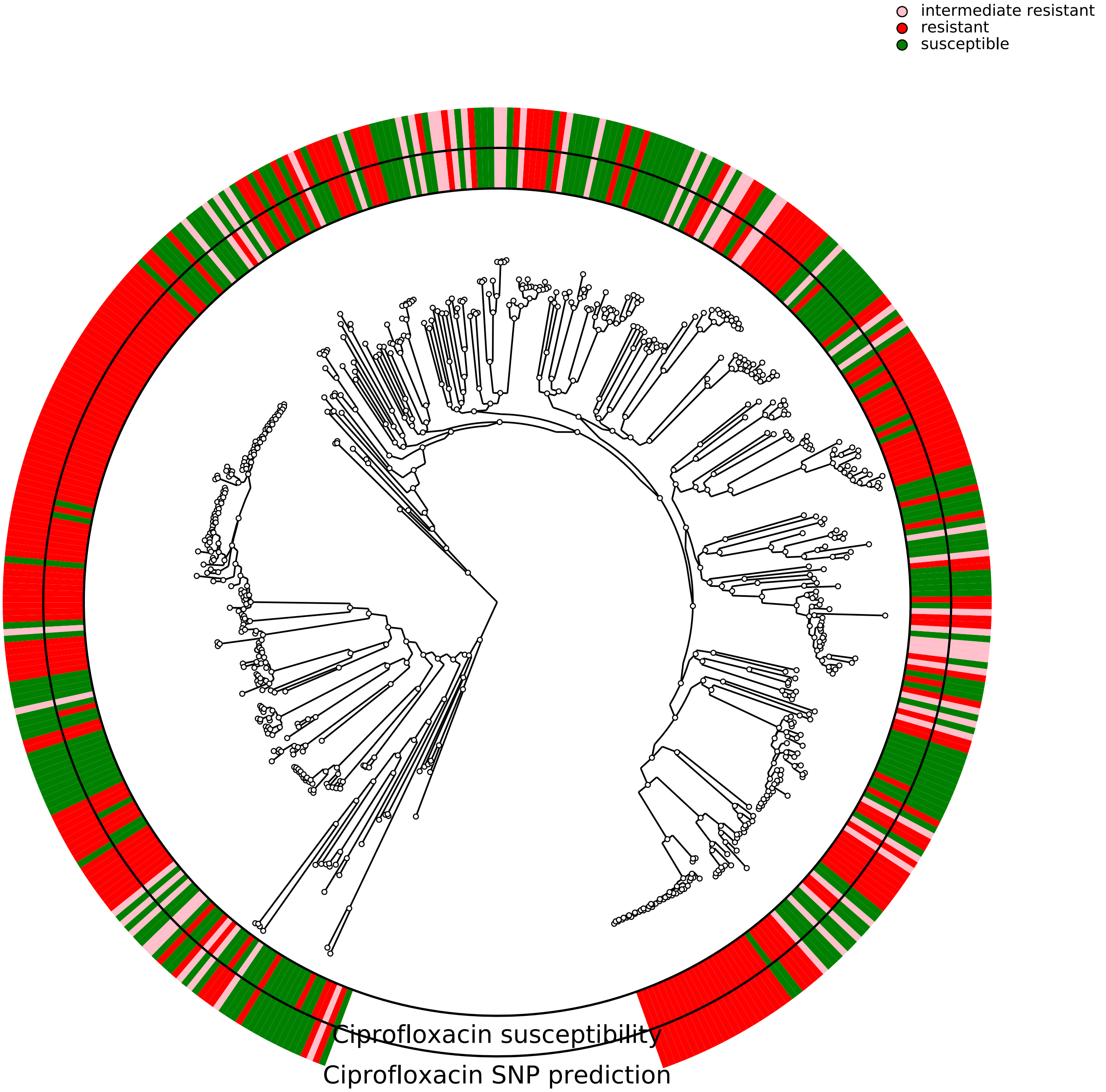

Figure EV2. Correlation of susceptibility profiling by MIC testing and resistance prediction using diagnostic classifiers for meropenem.

The phylogenetic tree is based on the 414 *Pseudomonas aeruginosa* isolates used in this study. The branches leading toward two deeply branching clades were collapsed (CH4433 and ESP077, and CH4684, CH5206, and CH5387). The inner ring depicts the susceptibility of each isolate to meropenem; green, susceptible; red, resistant; rose, intermediate resistant. The outer rings show the susceptibility as assigned by the diagnostic classifier (intermediate resistant samples were not assigned).

© 2020 The Authors

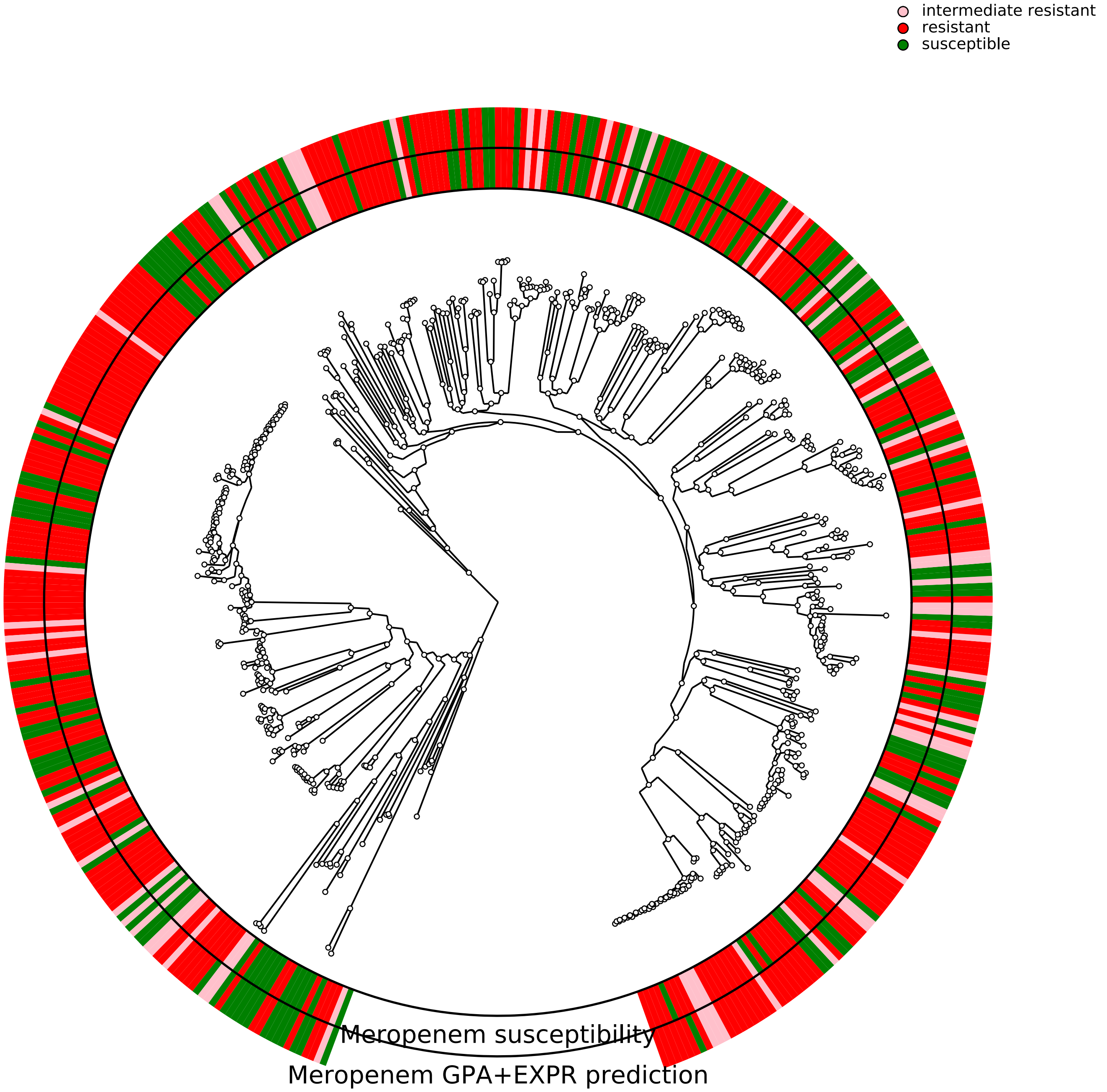

Figure EV3. Correlation of susceptibility profiling by MIC testing and resistance prediction using diagnostic classifiers for tobramycin.

The phylogenetic tree is based on the 414 *Pseudomonas aeruginosa* isolates used in this study. The branches leading toward two deeply branching clades were collapsed (CH4433 and ESP077, and CH4684, CH5206, and CH5387). The inner ring depicts the susceptibility of each isolate to tobramycin; green, susceptible; red, resistant; rose, intermediate resistant. The outer rings show the susceptibility as assigned by the diagnostic classifier (intermediate resistant samples were not assigned).

© 2020 The Authors

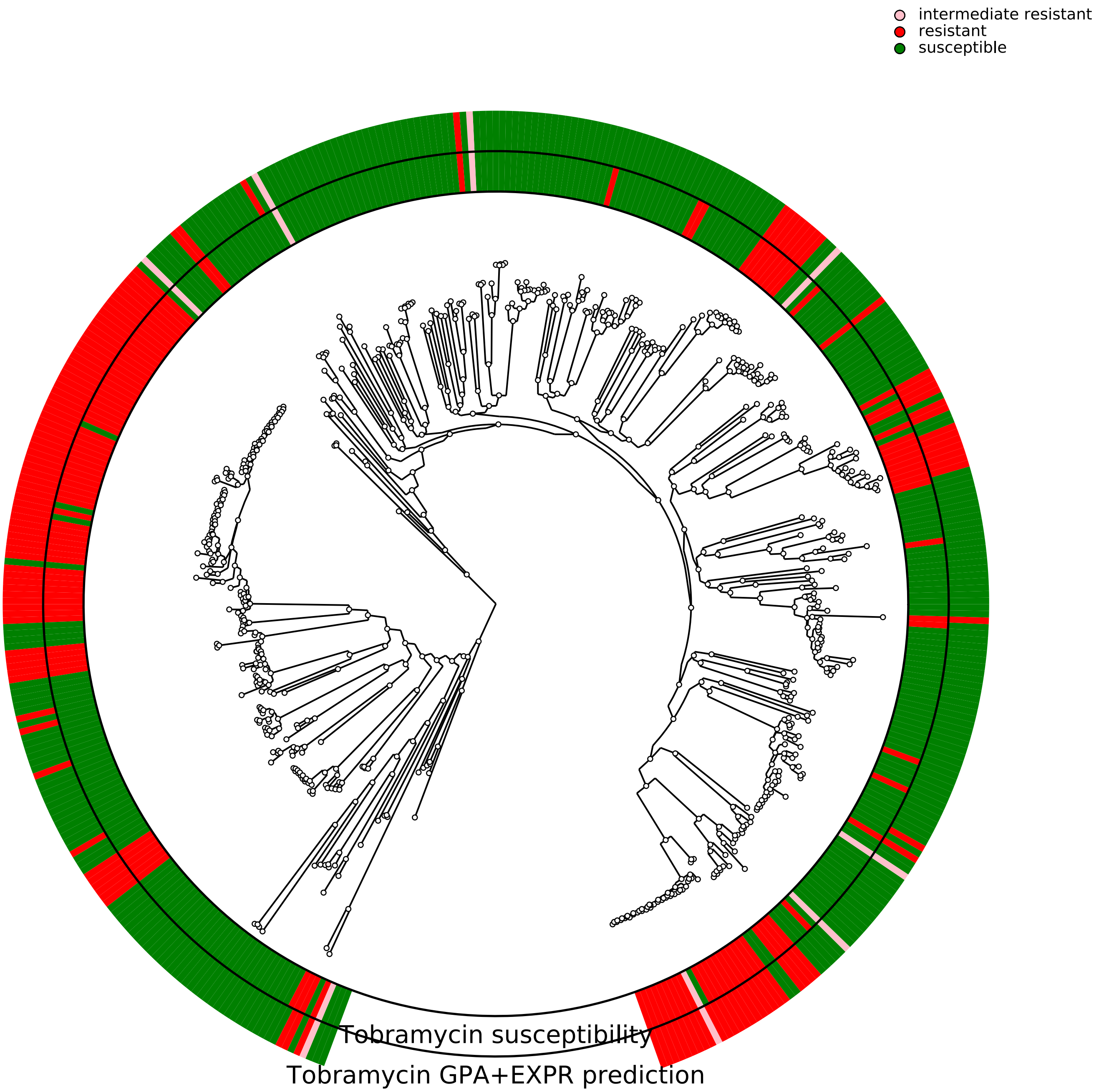

Figure EV4. Correlation of susceptibility profiling by MIC testing and resistance prediction using diagnostic classifiers for ceftazidime.

The phylogenetic tree is based on the 414 *Pseudomonas aeruginosa* isolates used in this study. The branches leading toward two deeply branching clades were collapsed (CH4433 and ESP077, and CH4684, CH5206, and CH5387). The inner ring depicts the susceptibility of each isolate to ceftazidime; green, susceptible; red, resistant; rose, intermediate resistant. The outer rings show the susceptibility as assigned by the diagnostic classifier (intermediate resistant samples were not assigned).

© 2020 The Authors

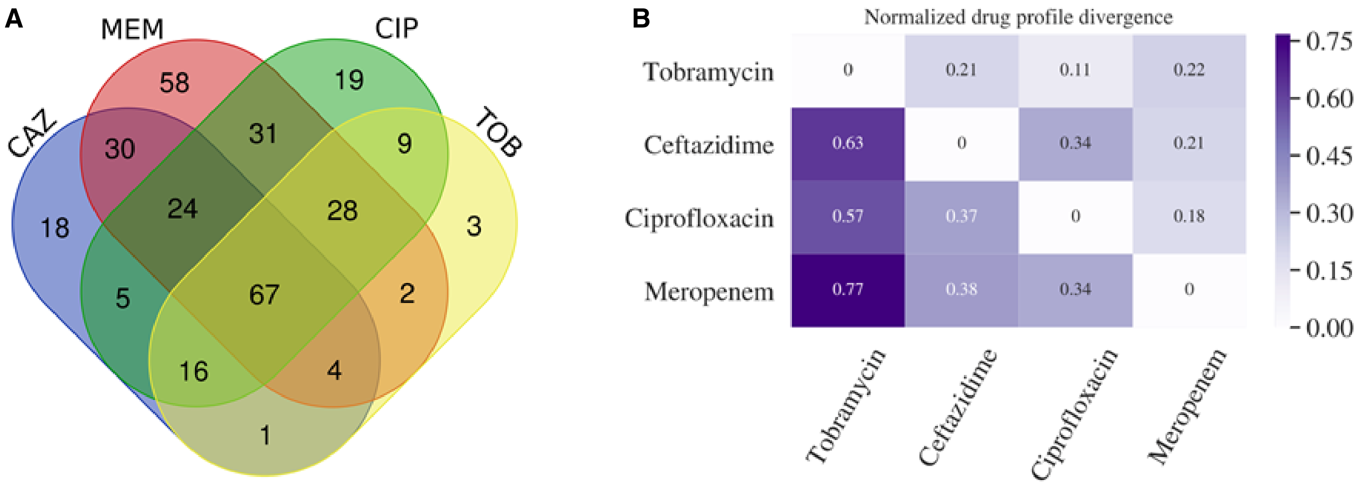

Figure EV5. Resistance overlap and correlation between different drugs using the Kullback–Leibler divergence of resistance profiles for different pairs of drugs.

A Venn diagram shows the overlap of resistances among the collected clinical isolates.

B To further investigate the co-resistance among the drugs, we also calculated the Kullback–Leibler divergence (KL divergence) between resistance patterns. We use the KL divergence as a non-symmetric measurement of the differences between resistance patterns of different drugs. We use a non-symmetric measure to be able to distinguish among the case of given a resistance to drug A, what does this imply for resistance against drug B (drug A, drug B) and the other way around (drug B, drug A). We normalized the KL values by dividing all values by the maximum in the table. In this analysis, the divergence is a measure to indicate whether information on a particular drug resistance could *not* be used to predict the simultaneous appearance of a second resistance. Thus, the higher the divergence value is, the less the information is available to predict a particular resistance pattern. The results imply, i.e., that TOB resistance comes with a higher probability of simultaneous MEM resistance, however, not the other way around.

 © 2020 The Authors
